# Waning of antibody levels induced by a 13-valent pneumococcal conjugate vaccine, using a 3 + 0 schedule, within the first year of life among children younger than 5 years in Blantyre, Malawi: an observational, population-level, serosurveillance study

**DOI:** 10.1016/S1473-3099(22)00438-8

**Published:** 2022-12

**Authors:** Todd D Swarthout, Marc Y R Henrion, Deus Thindwa, James E Meiring, Maurice Mbewe, Akuzike Kalizang’Oma, Comfort Brown, Jacquline Msefula, Brewster Moyo, Andrew A Mataya, Susanne Barnaba, Emma Pearce, Melita Gordon, David Goldblatt, Neil French, Robert S Heyderman

**Affiliations:** a National Institute for Health and Care Research Mucosal Pathogens Research Unit, Research Department of Infection, Division of Infection and Immunity, University College London, London, UK; b Malawi-Liverpool-Wellcome Research Programme, Blantyre, Malawi; c Department of Clinical Sciences, Liverpool School of Tropical Medicine, Liverpool, UK; d Centre for the Mathematical Modelling of Infectious Diseases, Department of Infectious Disease Epidemiology, London School of Hygiene and Tropical Medicine, London, UK; e Department of Infection, Immunity and Cardiovascular Disease, University of Sheffield, Sheffield, UK; f Faculty of Medicine, University of Amsterdam, Amsterdam, Netherlands; g Chancellor College, University of Malawi, Blantyre, Malawi; h Great Ormond Street Institute of Child Health, University College London, London, UK; i Institute of Infection, Veterinary and Ecological Sciences, University of Liverpool, Liverpool, UK

## Abstract

**Background:**

Pneumococcal conjugate vaccines (PCVs) induce serotype-specific IgG antibodies, effectively reducing vaccine-serotype carriage and invasive pneumococcal disease (IPD). IgG production wanes approximately 1 month after vaccination in absence of serotype-specific exposure. With uncertainty surrrounding correlate of protection (CoP) estimates and with persistent vaccine-serotype carriage and vaccine-serotype IPD after PCV13 introduction, we aimed to profile population-level immunogenicity among children younger than 5 years in Blantyre, Malawi.

**Methods:**

For this serosurveillance study, we used a random subset of samples from a prospective population-based serosurvey in Blantyre, Malawi, done between Dec 16, 2016, and June 27, 2018. Sample selection was based on age category optimisation among children younger than 5 years, adequate sample volume, and available budget. We measured serotype-specific IgGs against the 13 vaccine serotypes (1, 3, 4, 5, 6A, 6B, 7F, 9V, 14, 18C, 19A, 19F, and 23F) and two non-vaccine serotypes (12F and 33F), as well as IgGs against three pneumococcal proteins (PsaA, NanA, and Ply), using ELISA and a direct-binding electrochemiluminescence-based multiplex assay. We estimated population-level, serotype-specific immunogenicity profiles using a linear spline regression model. Analyses included samples stratified to 20 3-month age strata (eg, age <3 months to 57–59 months).

**Findings:**

We evaluated 638 plasma samples: 556 primary samples and 82 unique secondary samples (each linked to one primary sample). Immunogenicity profiles revealed a consistent pattern among vaccine serotypes except serotype 3: a vaccine-induced IgG peak followed by waning to a nadir and subsequent increase in titre. For serotype 3, we observed no apparent vaccine-induced increase. Heterogeneity in parameters included age range at post-vaccination nadir (from 11·2 months [19A] to 27·3 months [7F]). The age at peak IgG titre ranged from 2·69 months (5) to 6·64 months (14). Titres dropped below CoPs against IPD among nine vaccine serotypes (1, 3, 4, 5, 6B, 7F, 9V, 18C, and 23F) and below CoPs against carriage for ten vaccine serotypes (1, 4, 5, 6B, 7F, 9V, 14, 18C, 19F, and 23F). Increasing antibody concentrations among older children and seroincident events were consistent with ongoing vaccine-serotype exposure.

**Interpretation:**

A 3 + 0 PCV13 schedule with high uptake has not led to sustained population-level antibody immunity beyond the first year of life. Indeed, post-vaccine antibody concentrations dropped below putative CoPs for several vaccine serotypes, potentially contributing to persistent vaccine-serotype carriage and residual vaccine-serotype IPD in Malawi and other similar settings. Policy decisions should consider alternative vaccine strategies, including a booster dose, to achieve sustained vaccine-induced antibody titres, and thus control.

**Funding:**

Bill & Melinda Gates Foundation, Wellcome UK, and National Institute for Health and Care Research.

## Introduction

The encapsulated bacteria *Streptococcus pneumoniae* (pneumococcus) is responsible for approximately 300 000 deaths annually worldwide, with a third of these among children younger than 5 years and with the greatest burden occurring in low-income and middle-income countries (LMICs).^1^ *S pneumoniae* has almost 100 capsule serotypes, with 20–30 accounting for most invasive pneumococcal disease (IPD). Nasopharyngeal carriage of pneumococcus in the upper airway is a prerequisite for disease but is also a key process in the development of natural immunity.^2^ Immunocompromised individuals, infants, and older adults are at greater risk of IPD, with LMICs bearing the greatest burden.^3^, ^4^

**Research in context**

**Evidence before this study**

We implemented a targeted literature review strategy using PubMed, restricted from Jan 1, 2000, to Dec 31, 2021, incorporating the following search strategy: (((streptococcus pneumoniae[Title]) OR (pneumococc*[Title])) OR (antipneumococc*[Title])) AND (((((((immunogen*[Title]) OR (waning[Title])) OR (wane[Title])) OR (concentration*[Title])) OR (correlate*[Title])) OR (threshold*[Title])) OR (antibod*[Title])). Only one article presented population-level, serotype-specific immunogenic profiles, among Burmese refugees in Thailand before PCV introduction. These search results, together with the uncertainty surrounding CoP estimates, underlines the need to further evaluate heterogeneity in vaccine-induced IgG profiles and their relationship to pneumococcal colonisation and disease.

**Added value of this study**

We developed a novel linear spline regression model to define population-level serotype-specific immunogenicity profiles. In this under-5 population in Malawi, with a 3 + 0 PCV13 schedule and a catch-up campaign among children younger than 1 year, vaccine-induced antibody titres against multiple vaccine serotypes were not sustained at a population level beyond the first year of life. Indeed, IgG titres of several vaccine serotypes remained below putative CoPs against carriage and IPD for extended periods: titres of nine vaccine serotypes fell below the aggregate CoP against IPD (0·35 μg/mL) for a range of 0·2–51·9 months and below serotype-specific CoPs against carriage for all ten vaccine-serotypes evaluated (range 17·6–54·0 months). Antibody profiles were largely consistent across PCV13 vaccine serotypes except for serotype 3, with a vaccine-induced increase peaking at about age 5 months, subsequently falling to a nadir at about age 14 months, before increasing again. Our hypothesis that this post-nadir increase is due to accumulating natural exposure is supported by evidence of carriage of all 13 vaccine serotypes and seroincident events for carried serotypes.

**Implications of all the available evidence**

In the context of a 3 + 0 PCV13 schedule in a low-income country, we report the absence of a sustained vaccine-induced antibody response to multiple vaccine serotypes beyond the first year of life, potentially resulting in a window of vulnerability to carriage and disease. This is particularly relevant given that most sub-Saharan African countries implementing PCVs have used the same schedule of 3 primary doses with a limited catch-up campaign. With evidence of residual vaccine-serotype carriage and disease in settings similar to Malawi, strategies to achieve sustained vaccine-induced antibody titres are required. Policy decisions should favour including a booster dose later in the first or second year of life, as endorsed by WHO, to achieve sustained vaccine-induced antibody titres. By inducing higher antibody concentrations later in the child's life, a booster dose might lead to longer protection against colonisation and improved indirect protection, which is crucial for successful PCV programmes.

Pneumococcal conjugate vaccines (PCVs), currently including ten (PCV10) or 13 (PCV13) capsular polysaccharides, have been introduced into routine infant immunisation programmes across the world. These vaccines have been effective in reducing nasopharyngeal carriage prevalence and IPD incidence among both vaccinated (direct protection) and unvaccinated (indirect protection) children and adults.^5^, ^6^ PCVs elicit protection against disease in infants through induction of serotype-specific opsonophagocytic antibodies.^7^ IgG antibody production induced by PCV peaks approximately 1 month after vaccination and wanes thereafter in the absence of serotype-specific natural exposure.^8^ A pooled serum IgG correlate of protection (CoP) against IPD of 0·35 μg/mL was derived from a meta-analysis of three efficacy trials.^9^ Although the use of 0·35 μg/mL as an aggregate CoP has enabled the licensing of new PCVs, a post licensure study subsequently reported serotype-specific CoPs that vary widely, with some higher (1, 3, 7F, 19A, and 19F) and others lower (6A, 6B, 18C, and 23F) than 0·35 μg/mL (appendix p 3).^10^

A CoP against pneumococcal carriage has been more problematic to derive, partly because of the complexity of interactions between pneumococcus and the human mucosal immune system.^11^ Here, serotype-specific effector mechanisms probably include both circulating serum IgG and locally produced IgA and IgG in the context of T-cell immunity to subcapsular protein antigens and cellular and soluble innate immunity.^12^, ^13^ To overcome considerable heterogeneity, some studies used data from multiple vaccine immunogenicity trials to calculate serotype-specific carriage CoPs, reporting estimates that are higher for carriage than for IPD and are serotype-specific, with CoP against 6B among the lowest (0·50 μg/mL) and that against 19F among the highest (2·54 μg/mL; appendix p 3).^14^, ^15^ Importantly, the studies also found that protective correlates were, on average, two-times higher in LMICs than in high-income countries.

Malawi introduced PCV13 into the national Expanded Programme of Immunisations in 2011, using a 3 + 0 schedule (one dose given at 6, 10, and 14 weeks of age) with a catch-up campaign limited to children aged younger than 1 year. Field studies reported high and timely vaccine coverage, exceeding 90%.^16^, ^17^ Although we have shown reduced vaccine-serotype carriage in Malawi, we saw persistent residual carriage of all 13 vaccine serotypes among children vaccinated with PCV13 up to 7 years after PCV13 introduction.^17^ Similar patterns have been seen in Kenya^18^ and The Gambia.^19^ We also showed that, despite a vaccine-attributable reduction in vaccine-serotype IPD among children of eligible age for vaccination, residual vaccine-serotype IPD persisted among both PCV-vaccinated individuals and those who had not received a PCV vaccine.^20^ This suggests suboptimal control of colonisation and onward transmission, as well as persistent vulnerability to vaccine-serotype IPD, even among children vaccinated with PCV13.

We have suggested that a high force of infection in settings such as Malawi contributes to a short duration of PCV13-induced vaccine-serotype carriage control,^21^ hypothesising that the rapid waning of vaccine-induced antibodies resulting from the 3 + 0 schedule enables persistent vaccine-serotype carriage. To further evaluate the complex interaction between immunity (vaccine-induced and through natural exposure) and colonisation, we aimed to undertake a population-based pneumococcal serology profiling among children younger than 5 years in Blantyre, Malawi, to describe the serotype-specific IgG profiles of these children and assess their effect on carriage acquisition and IPD among key PCV13 serotypes using putative serological CoPs.

## Methods

### Study design and participants

Malawi is a low-income country in southern Africa. Blantyre (population 1·3 million) is the geographical centre of the country's Southern Region. A series of rolling pneumococcal carriage surveys (the PCVPA study) were implemented between 2015 and 2019 in the urban townships of Blantyre city, randomly sampling healthy participants, including children aged 18 weeks to 7 years (among those vaccinated with PCV13) and children aged 3–10 years (among those age-ineligible to receive PCV13).^17^ Despite evidence of reduced vaccine-serotype carriage, we identified persistent residual carriage of all 13 vaccine serotypes up to 7 years after PCV13 introduction.^17^ Vaccine-serotype carriage prevalence among children from the PCVPA study is presented in the appendix (p 4).

Our study is based on a random subset of samples from a prospective population-based serosurvey (STRATAA study) in Ndirande, a large urban township of Blantyre city. This survey was done between Dec 16, 2016, and June 27, 2018, to understand the burden of enteric fever.^23^, ^24^ For STRATAA study recruitment, a subset of 8500 Ndirande residents were randomly selected from a population demographic census and invited to participate. The vaccination status of child participants was obtained from patient-retained health books (known as health passports) or, if documentation was unavailable, from parents or guardians. All STRAATA study participants were followed up to provide a linked secondary sample approximately 3 months after the first sample. For this evaluation of pneumococcal serology, we based sample selection on optimising an equal distribution across age categories among children younger than 5 years, having adequate sample volume for the serological assays, and available budget. Samples were randomly selected. To better understand the influence of seroincident events (ie, pneumococcal acquisition due to natural exposure) on serotype-specific antibody titres, we included secondary samples that were each linked to a unique primary sample. The dynamics of antibody decay was based on a previously published work.^25^

The work received ethics approval from the Oxford Tropical Research Ethics Committee (reference 39–15), the Malawian National Health Sciences Research Committee (15/11/1511), and the Kamuzu University of Health Sciences (formerly College of Medicine, University of Malawi) Research and Ethics Committee (P.02/15/1677). Parents or guardians of child participants provided written informed consent, including consent for publication.

### Procedures

Venous plasma samples treated with EDTA were stored in cool boxes upon collection and transported to the study laboratory. Plasma was separated by centrifugation and stored at −80°C within 8 h of collection.

Serological analysis was done at the WHO reference laboratory for pneumococcal serology, Great Ormond Street Institute of Child Health, University College London (London, UK). Plasma was stored at –70°C before the assay for serotype-specific IgG against capsular polysaccharides of the 13 vaccine serotypes (1, 3, 4, 5, 6A, 6B, 7F, 9V, 14, 18C, 19A, 19F, and 23F) and two non-vaccine serotypes (12F and 33F). Serotypes 12F and 33F, serving in effect as negative controls, were selected among other non-vaccine serotypes because they were confirmed to be present in carriage in Malawi and they are included in the recently approved 15-valent PCV (Vaxneuvance; Merck Sharp & Dohme, Rahway, NJ, USA) and 20-valent PCV (Prevnar 20; Wyeth Pharmaceuticals, Madison, NJ, USA). We assayed plasma samples using the WHO reference ELISA following adsorption with cell wall polysaccharide (SSI Diagnostica, Hillerød, Denmark) and 22F polysaccharide (American Type Culture Collection, Manassas, VA, USA) at a concentration of 10 μg/mL as previously described.^26^ This assay is based on the original Wyeth assay used to generate the aggregate CoP of 0·35 μg/mL. The lower limit of assay quantification was 0·15 μg/mL. We measured serum IgG against pneumococcal protein (including PsaA, NanA, and Ply) antigens using a direct binding electrochemiluminescence-based multiplex assay (MesoScale Discovery; Rockville, MD, USA) on customised antigen plates as previously described.^27^

### Statistical analysis

A sample size of 556 allowed for predicting the time, after vaccination, when 50% of the population would fall below a certain anti-capsular antibody level (0·35 μg/mL and 1·0 μg/mL) with a 95% precision of 8 weeks (ie, plus or minus 4 weeks around the point estimate).

We summarised participant demographic characteristics using the median for continuous variables. We compared covariate distribution between age groups using *t* test for continuous covariates and χ^2^ for categorical covariates. Statistical significance was inferred from two-sided p<0·05.

The relationship between age and IgG is nonlinear and changes with age. To model the nonlinear relationship between age and IgG with generalised linear regression models and to perform statistical inference on the locations of the changepoints, we used linear splines. The regression models we developed are piecewise linear models, joined at specific values of the predictor variable. These locations, referred to as changepoints or knots, define the intervals over which the relationship between predictor and response is assumed to be linear. The assay's lower limit of detection (0·15 μg/mL) means that observations below the detection limit were left-censored. To account for this, we used censored regression. As a prerequisite, the fitted models needed to satisfy several requirements, including the following: to properly account for data values that are left-censored (below the assay detection limit), to treat the knot locations as parameter values to be estimated during model fitting, to return a fit for the geometric mean IgG concentration as a function of age, and to select the optimal number of changepoints. To achieve this, we fitted censored regression models with linear splines for the age predictor variable, treating knot locations as additional model parameters, and we manually optimised the resulting likelihood function. We used bootstrapping and the percentile method to derive confidence intervals for slope parameters and knot locations. Before model fitting, the IgG data were log-transformed so that the fitted arithmetic mean corresponded with the log of the geometric mean on the original data scale. For every serotype, we fitted models with k=0, 1, 2, 3 knots and then selected the optimal value for k using the Akaike information criterion. Implementation was done using R, version 4.1.2. The modelling code is available on GitHub.^28^ Further details on the modelling methods are provided in the appendix (pp 5–6).

### Role of the funding source

The study funders had no role in study design, data collection, data analysis, data interpretation, decision to submit for publication, or writing of the report.

## Results

We evaluated 638 plasma samples for serotype-specific IgG titres among children younger than 5 years, including 556 primary samples and 82 unique secondary samples (each linked to one primary sample). Among 547 participants with available gender data, 279 (51%) were boys, and the median age at primary sample collection was 1·9 years (range 42 days to 4·9 years, IQR 1·1–3·3 years; table 1). Secondary plasma samples were collected approximately 3 months (median 98 days, range 83–107) after the primary sample.

**Table 1:** Participant characteristics by age group

	**Gender**		**Household size**	**Primary caregiver completed secondary education**	**Primary caregiver is employed**
	Boys	Girls			
<3 months	7 (64%; n=11)	4 (36%; n=11)	6 (4–9)	3 (50%; n=6)	6 (100%; n=6)
3–5 months	8 (53%; n=15)	7 (47%; n=15)	4 (4–5)	3 (30%; n=10)	10 (100%; n=10)
6–11 months	43 (48%; n=89)	46 (52%; n=89)	5 (4–6)	25 (43%; n=58)	56 (97%; n=58)
1 year	96 (51%; n=188)	92 (49%; n=188)	5 (4–6)	61 (48%; n=127)	112 (88%; n=127)
2 years	46 (55%; n=84)	38 (45%; n=84)	5 (4–6)	19 (40%; n=48)	44 (92%; n=48)
3 years	33 (42%; n=78)	45 (58%; n=78)	5 (4–6)	19 (40%; n=48)	44 (92%; n=48)
4 years	46 (56%; n=82)	36 (44%; n=82)	5 (4–7)	21 (40%; n=53)	47 (89%; n=53)
Total	279 (51%; n=547)	268 (49%; n=547)	5 (4–6)	151 (46%; n=330)	319 (91%; n=350)

Data are n (%; N) or median (IQR). Analysis is based on available data from 556 primary samples. There were no significant differences between age strata.

The 556 primary samples were stratified to 20 3-month age strata (eg, aged <3 months to 57–59 months), with a median 21 samples (range nine to 54) per stratum (appendix pp 7–8). 373 (67%) of the children had health passports (patient-retained health records). Based on either health passport or information from guardians, 545 (98%) of children completed three-dose PCV13 vaccination, with all children having received at least one timely dose (ie, within 3 weeks of their schedule visit). Those receiving fewer than three doses were among the youngest age group and age-ineligible. In comparing age strata, the distribution of participant characteristics was similar, with no significant differences between age strata (table 1).

The number of serology results per serotype is shown in the appendix (p 9). In brief, a range of 599 (serotype 3) to 637 (serotype 6A) samples provided serotype-specific IgG concentration data included in the analysis.

**Figure 1:**
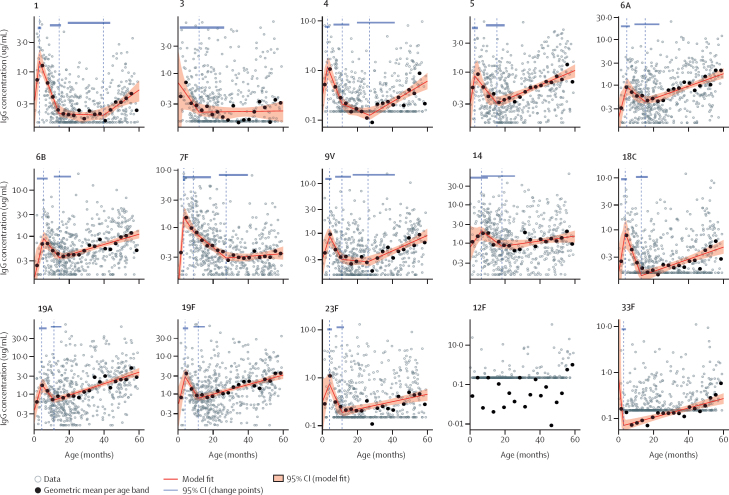
Linear spline regression model evaluating the relationship between age and IgG Grey hollow dots indicate empirical IgG titre datapoints for each sample. Black dots are the geometric mean concentrations for all datapoints within each 3-month age band. The red line is the spline model fit and the shaded area is the 95% CI for the model fit. Vertical dashed lines indicate changepoints. Blue bars at the top of each image are the 95% CI for the age at each changepoint. Y-axes differ in scale, including IgG concentrations from 0–1 μg/mL (serotype 12F), 0–3 μg/mL (serotypes 1, 3, and 5), 0–10 μg/mL (serotypes 4, 6B, 7F, 9V, 18C, 23F, and 33F), and 0–30 μg/mL (serotypes 6A, 14, 19A, and 19F). The row of grey dots at the bottom edge of each image are datapoints with titres below the lower limit of detection. This analysis took the actual lower limit of detection into account and treated all values of 0·15 μg/mL or lower as left-censored. Y-axis slope parameters are on the log-IgG scale. The values for each calculated geometric mean concentration (95% CI) are presented in the appendix (pp 7–8).

The population-level spline model output, based on 556 primary samples (figure 1, table 2), showed a consistent pattern among all vaccine serotypes, except serotype 3. We observed a period of lower titres among young infants, reflecting infants with fewer than three PCV13 doses and probably detection of maternal antibodies. This period was followed by a vaccine-induced increase (positive slope) in IgG until a peak titre, a period of waning (negative slope) until a nadir titre, and a subsequent increase in titre, which might be due to accumulating natural exposure to *S pneumoniae*. Serotype 3 showed no population-level vaccine-induced increase and, unique to serotype 3, no post-nadir increase (slope=0), despite evidence of natural exposure (appendix p 4). We observed no vaccine-induced increase in IgG titres of non-vaccine serotype 33F, although a post-nadir increase was observed. No model could be fitted for non-vaccine serotype 12F because too many measurements were either below the assay's lower limit of detection (467 [73%] of 638) or had no detectable IgG (108 [17%]). Three serotypes were associated with a two-stage population-level waning after the post-vaccine peak, with steeper initial waning and later nadirs than those of other serotypes (including reaching nadir at ages 26·1 months [9V], 27·0 months [4], and 27·3 months [7F]). Serotype 1 included a long post-nadir trough (period of no change in population-level titre [slope=0]) between ages 14·1 and 39·5 months before an increase in titre was detected. It should be noted that the confidence intervals of several slope estimates in table 2 include 0 or a negative slope value (eg, post-nadir slopes of serotypes of 7F and 14).

**Table 2:** Serotype specific parameter estimate from a population-level spline model analysis

	**Profile**	Increase in IgG,*vaccine induced			**Waning IgG**†			**Increase in IgG, natural exposure**	
		Slope to peak	Age at peak, months	IgG concentration at peak, μg/mL	Slope to nadir‡	Age at nadir, months	IgG concentration at nadir, μg/mL	Slope	IgG concentration at final, μg/mL
1	2	0·55 (0·50 to 0·82)	2·80 (1·86 to 3·81)	1·5 (0·75 to 2·25)	−0·17 (−0·30 to −0·14)	14·10 (8·82 to 15·14)	0·20 (0·17 to 0·26)	0·05 (0·02 to 0·07)	0·51 (0·33 to 0·72)
3	1	−0·10 (−0·40 to 10·76)	NA	NA	0·00 (−0·01 to 0·02)	12·00 (1·03 to 26·30)	0·22 (0·19 to 0·31)	NA	0·23 (0·14 to 0·34)
4	3	0·46 (0·38 to 0·72)	3·07 (1·59 to 4·27)	1·18 (0·55 to 1·77)	S2 −0·21 (−0·34 to −0·13); S3 −0·03 (−0·09 to 0·02)§	27·02 (19·83 to 41·36)	0·13 (0·10 to 0·18)	0·05 (0·03 to 0·07)	0·61 (0·42 to 0·90)
5	4	0·41 (0·14 to 0·96)	2·69 (1·08 to 4·43)	0·86 (0·51 to 1·36)	−0·08 (−0·27 to −0·05)	15·34 (9·11 to 19·76)	0·31 (0·29 to 0·40)	0·03 (0·02 to 0·04)	1·07 (0·82 to 1·34)
6A	4	0·33 (0·20 to 0·63)	4·71 (1·37 to 6·48)	0·91 (0·55 to 1·27)	−0·07 (−0·24 to −0·02)	15·25 (9·11 to 23·30)	0·44 (0·39 to 0·58)	0·03 (0·02 to 0·04)	1·78 (1·39 to 2·34)
6B	4	0·38 (0·19 to 0·59)	5·24 (1·45 to 7·57)	0·90 (0·47 to 1·25)	−0·11 (−0·27 to −0·03)	14·25 (11·11 to 20·94)	0·35 (0·32 to 0·47)	0·03 (0·02 to 0·04)	1·12 (0·91 to 1·41)
7F	3	0·88 (0·72 to 1·06)	3·12 (2·62 to 3·90)	1·47 (0·82 to 2·13)	S2 −0·10 (−0·23 to −0·04); S3 −0·06 (−0·07 to −0·02)§	27·33 (23·21 to 39·78)	0·29 (0·27 to 0·38)	0·01 (−0·01 to 0·03)	0·35 (0·27 to 0·46)
9V	3	0·26 (0·19 to 0·48)	4·19 (1·77 to 5·33)	0·88 (0·56 to 1·23)	S2 −0·15 (−0·31 to −0·07); S3 −0·01 (−0·05 to 0·03)§	26·14 (17·39 to 41·44)	0·29 (0·23 to 0·35)	0·04 (0·02 to 0·06)	0·91 (0·70 to 1·24)
14	4	0·15 (−0·01 to 0·39)	6·64 (2·86 to 10·30)	2·07 (1·16 to 2·63)	−0·08 (−0·22 to −0·01)	18·35 (6·93 to 25·79)	0·82 (0·72 to 1·21)	0·01 (0·00 to 0·03)	1·51 (1·02 to 2·10)
18C	4	0·47 (0·12 to 1·14)	3·78 (1·45 to 4·76)	0·87 (0·43 to 1·42)	−0·21 (−0·36 to −0·10)	12·95 (9·89 to 16·67)	0·13 (0·11 to 0·21)	0·03 (0·02 to 0·04)	0·47 (0·35 to 0·64)
19A	4	0·38 (0·17 to 0·57)	4·22 (2·79 to 6·90)	1·81 (1·01 to 2·46)	−0·14 (−0·34 to −0·05)	11·24 (9·47 to 15·48)	0·70 (0·60 to 0·97)	0·04 (0·03 to 004)	3·86 (3·06 to 4·96)
19F	4	0·45 (0·16 to 0·80)	3·79 (2·58 to 5·39)	2·88 (1·38 to 4·38)	−0·17 (−0·42 to −0·10)	11·34 (8·61 to 15·12)	0·78 (0·67 to 1·10)	0·03 (0·02 to 0·04)	3·17 (2·51 to 4·01)
23F	4	0·21 (−0·02 to 0·67)	4·17 (2·96 to 5·59)	0·74 (0·46 to 1·25)	−0·19 (−0·53 to −0·15)	11·34 (8·22 to 12·46)	0·19 (0·16 to 0·25)	0·02 (0·01 to 0·03)	0·45 (0·33 to 0·58)
12F¶	1	NA	NA	NA	NA	NA	NA	NA	0·27 (0·21 to 0·35)
33F	1	NA	No peak	No peak	NA	2·90 (1·76 to 4·17)	0·07 (0·06 to 0·14)	NA	NA

Data are estimate (95% CI). Profiles are visually defined (ie, not based on criteria of statistical significance) to help the reader categorise trends: 1 indicates that the fitted model estimated no vaccine-induced IgG increase; 2 indicates that the fitted model estimated a trough after the nadir for serotype 1, indicating no change in IgG titres from age 14·0 to 39·5 months; 3 indicates that the fitted model estimated two unique slopes within the waning phase; and 4 indicates that the fitted model estimated two changepoints—one at IgG peak and one at nadir before an increase in titre presumably due to natural exposure. NA=not applicable. S2=second slope estimated by the linear spline model. S3=third slope estimated by the linear spline model.

* Although increase in IgG can include IgG induced by natural exposure, we hypothesise that this stage is driven largely by vaccine-induced IgG.

† The aggregate dynamic of waning IgG will be influenced by waning of maternal and vaccine-induced IgG and increased IgG induced by natural exposure.

‡ Nadir refers to the minimum population-level IgG titre estimated.

§ Both S2 and S3 values occur during post-vaccination waning.

¶ No model could be fitted for serotype 12F as almost all measurements were below the detection limit.

The age at peak IgG (after starting the vaccination series) ranged from 2·7 months (serotype 5) to 6·6 months (serotype 14; table 2). The age at nadir (after vaccine-induced peak) ranged from 11·2 months (19A) to 27·3 months (7F). The IgG titres at nadir ranged from 0·13 μg/mL (4 and 18C) to 0·82 μg/mL (14). These changepoints (peaks and nadirs) were based on population-based data where the sampling was not done at the same time after vaccination and with varying waning of maternal antibodies. Therefore, there was some variance in the precision surrounding the peak.

An analysis of the 82 pairs of linked samples, censored to include only children older than 6 months, showed evidence of seroincident events (ie, a higher IgG titre in the secondary sample than in the primary sample) during follow-up (appendix pp 10–11). Serotype 5, for example, had the highest proportion of seroincident events, with 36 (49%) of 74 linked sample pairs indicating an increase in IgG titre: eight pairs (11%) with a two-fold increase or higher and three (4%) with a four-fold increase or higher. Evidence of increasing incidence of exposure events with age was further supported by NanA and Ply protein-specific antibody profiles (appendix p 12). Protein responses started increasing at roughly the same age as with post-nadir anti-capsular IgG, with similar trends reported in another study.^27^

**Figure 2:**
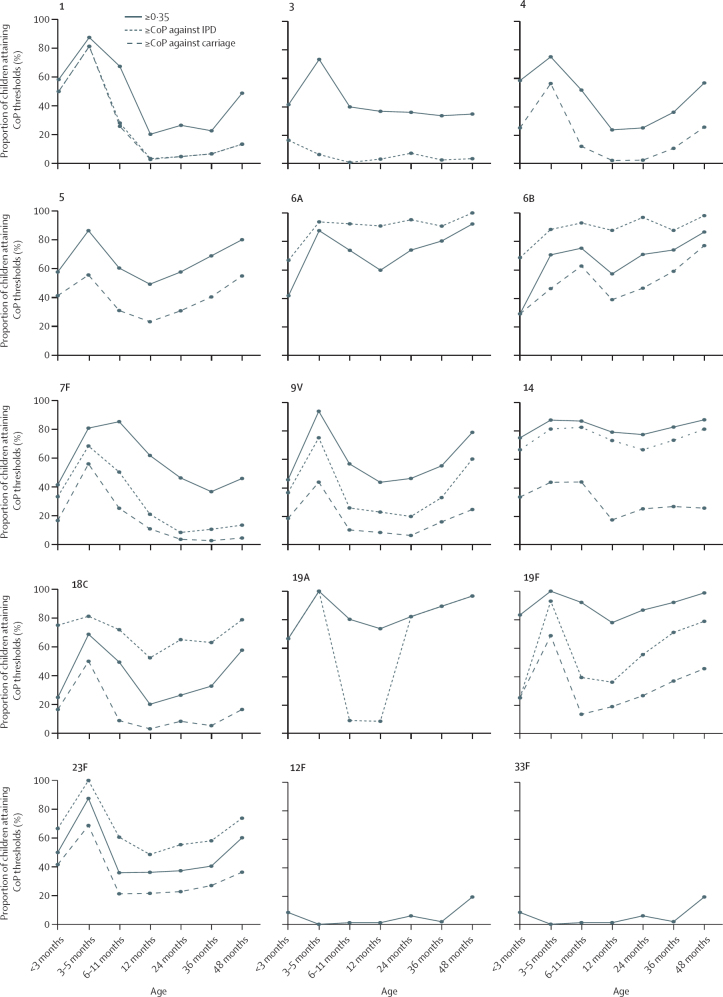
Percentage of children attaining CoP thresholds after PCV13 vaccination, stratified by age group Solid lines indicate the percentage of children with a serotype-specific IgG concentration exceeding 0**·**35 μg/mL. Dotted lines indicate the percentage of children with a serotype-specific IgG concentration exceeding the putative CoP against IPD^10^: 0·78 μg/mL for serotype 1, 3=2·83 μg/mL for serotype 3, 0·35 μg/mL for serotype 4, undefined for serotype 5, 0·16 μg/mL for serotype 6A, 0·16 μg/mL for serotype 6B, 0·87 μg/mL for serotype 7F, 0·62 μg/mL for serotype 9V, 0·46 μg/mL for serotype 14, 0·14 μg/mL for serotype 18C, 1·00 μg/mL for serotype 19A, 1·17 μg/mL for serotype 19F, and 0·20 μg/mL for serotype 23F. Dashed lines indicate the percentage of children with a serotype-specific IgG concentration exceeding the putative CoP against carriage^14^: 0·81 μg/mL for serotype 1, undefined for 3, 1·16 μg/mL for 4, 0·73 μg/mL for 5, undefined for 6A, 0·50 μg/mL for 6B, 1·60 μg/mL for 7F, 1·31 μg/mL for 9V, 2·48 μg/mL for 14, 1·32 μg/mL for 18C, undefined for 19A, 2·54 μg/mL for 19F, and 0·63 μg/mL for 23F. Note that some serotypes (1, 3, 4, 5, 6A, 19A, 12F, and 33F) have no putative CoP data for IPD or carriage, reflected by having fewer lines. The values used to develop this figure are presented in the appendix (p 13). CoP=correlate of protection. IPD=invasive pneumococcal disease.

Figure 2 shows the proportions of samples attaining putative CoPs associated with prevention of IPD and carriage. The data used to develop this figure are reported in the appendix (p 13). CoPs associated with preventing disease included 0·35 μg/mL and serotype-specific CoPs previously reported.^10^ CoPs associated with preventing carriage included serotype-specific CoPs previously reported.^14^ Except for serotypes 7F (lowest rate of increase [slope=0·01] in post-nadir IgG concentrations) and serotype 3, the proportion of children attaining the aggregate 0·35 μg/mL CoP against IPD, for example, dropped until approximately reaching the 1-year age group, increasing thereafter until the 4-year age group. With the exception of serotype 3, we observed a similar population trend for all CoP thresholds evaluated.

**Figure 3:**
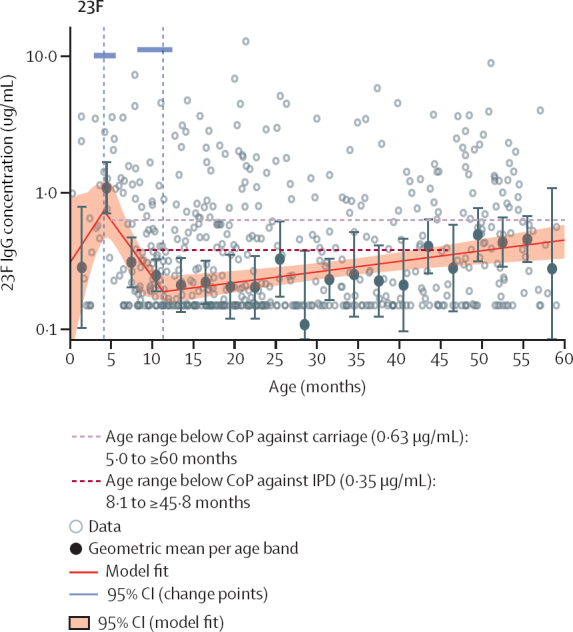
Population anti-23F titre trends and age periods when IgG titre drops below putative CoPs Spline regression model output for vaccine serotype 23F with age periods indicated for when the population-level model fit falls below putative CoPs. Grey hollow dots indicate empirical IgG titre datapoints for each sample. Black dots are the geometric mean concentrations for all datapoints within each 3-month age band. The red line is the spline model fit and the shaded area is the 95% CIs for the model fit. Vertical dashed lines indicate changepoints. Blue bars at the top of each image are the 95% CIs for the age at each changepoint. In this figure, the model fit for anti-23F antibody titres was estimated to be below the CoP against carriage (0·63 μg/mL) from age 5·0 months to 60 months or older (ie, beyond the age included in this analysis) and below the CoP against IPD (0·35 μg/mL) from age 8·1 months to 45·8 months. Table 3 shows estimated age periods when the model fit falls below the putative carriage and IPD thresholds for other vaccine-serotypes. CoP=correlate of protection. IPD=invasive pneumococcal disease.

By aligning our serotype-specific immunogenic profiles with putative CoPs, we estimated population-level titres that dropped below IPD and carriage CoPs for extended periods before cumulative natural exposure induced an increase in titres (table 3). Population-level titres of nine vaccine serotypes fell below 0·35 μg/mL for an age range of 0·2–51·9 months (median 22·9). The titres of nine vaccine-serotypes fell below the serotype-specific CoP for IPD for an age range of 5·0–52·0 months (24·8). For available carriage CoPs, titres fell below the serotype-specific CoP for all ten vaccine serotypes previously reported^14^ (range 17·6–54·0 months, 55·4). Figure 3 shows the spline model output for 23F, as an example, with ages with titres below the CoPs indicated.

**Table 3:** Estimated age range after vaccine-induced peak when IgG titres are below the CoPs for invasive pneumococcal disease and carriage among children younger than 5 years

	**Invasive pneumococcal disease**		**Age range below serotype-specific CoP for carriage**†
	Age range below 0·35 μg/mL, months	Age range below serotype-specific CoP for disease, months*	
1	11·1–52·0	7·0–≥59	6·3–≥59
3	7·1–≥59‡	All ages <60‡	UND
4	9·0–48·4	10·5–44·0	3·0–≥59
5	13·9–19·7	UND	4·8–46·3
6A	No ages <60	No ages <60	UND
6B	14·2–14·4	No ages <60	10·8–28·4
7F	24·2–≥59	9·5–≥59	All ages <60
9V	10·3–33·2	6·0–48·5	All ages <60
14	No ages <59	No ages <60	All ages <60
18C	8·2–49·3	12·0–22·5	All ages <60
19A	No ages <60	7·5–21·5	UND
19F	No ages <60	10·0–26·0	4·6–≥59
23F	8·1–45·8	11·0–16·0	5·0–≥59

CoP=correlate of protection. UND=undefined.

* Putative CoP for invasive pneumococcal disease^10^: 0·78 μg/mL for serotype 1, 2·83 μg/mL for 3, 0·35 μg/mL for 4, undefined for 5, 0·16 μg/mL for 6A, 0·16 μg/mL for 6B, 0·87 μg/mL for 7F, 0·62 μg/mL for 9V, 0·46 μg/mL for 14, 0·14 μg/mL for 18C, 1·00 μg/mL for 19A, 1·17 μg/mL 19F, and 0·20 μg/mL 23F.

† Putative CoP for carriage^14^ limited to serotypes of the ten-valent pneumococcal conjugate vaccine: 0·81 μg/mL for serotype 1, undefined for 3, 1·16 μg/mL for 4, 0·73 μg/mL for 5, undefined for 6A, 0·50 μg/mL for 6B, 1·60 μg/mL for 7F, 1·31 μg/mL for 9V, 2·48 μg/mL for 14, 1·32 μg/mL for 18C, undefined for 19A, 2·54 μg/mL for 19F, and 0·63 μg/mL for 23F.

‡ Model estimates no vaccine-induced increase in anti-serotype 3 antibody.

## Discussion

In this under-5 population in Malawi with a 3 + 0 PCV13 vaccine schedule with a catch-up campaign among children younger than 1 year, vaccine-induced antibodies against multiple vaccine serotypes were not sustained at the population level beyond the first year of life. Despite a post-nadir increase, presumably due to natural exposure, antibody concentrations of several vaccine-serotypes remained below putative CoPs for both IPD and carriage for extended periods. This probably contributes to the previously reported residual circulation of vaccine serotypes and vaccine-serotype IPD.^17^, ^20^ Indeed, the absence of population immunity against serotype 3 (a commonly carried vaccine serotype) and the extended period during which immunity against serotype 1 (uncommonly carried, but a prominent cause of disease) fell below the CoPs for IPD and carriage after the first year of life are of particular concern.

This population-level analysis of age-associated anti-capsular immunity reveals antibody profiles that are largely consistent across vaccine serotypes, except for serotype 3. We observed a vaccine-induced increase in IgG that peaks at about age 5 months (range 2·7–6·6) for 12 serotypes that, according to several efficacy trials and post-introduction effectiveness studies, is likely to be protective.^5^, ^29^ However, this peak is not sustained, falling below the putative IPD CoP (0·35 μg/mL), for example, for nine vaccine serotypes at about age 11 months (range 7·1–24·2). Our hypothesis that the post-nadir increase in titres is due to accumulating natural exposure is supported by evidence of persistent carriage of all 13 vaccine serotypes and consequent seroincident events for commonly carried serotypes. The heterogeneity between individual vaccine serotypes in the rates of vaccine-induced IgG production and waning in relation to the control of pneumococcal colonisation has received insufficient attention to date.

Serotype-specific immunogenic profiles among infants are complex, comprising a combination of waning maternal antibodies and antibodies induced by both vaccines and natural exposure. Previous studies have shown varying prevalences of maternal pneumococcal antibodies, for example, from highest for serotypes 14 (92%) and 19F (80%) to lowest for serotypes 4 (30%) and 1 (34%).^30^

For serotype 3, we saw no evidence of a population-level vaccine-induced antibody and, despite a relatively high prevalence of carriage, no post-nadir increase up to age 59 months. Unique to serotype 3, these findings are consistent with previous evidence that PCV13 confers less and shorter-term protection against serotype 3 among vaccinated children compared with other vaccine serotypes.^31^ We should note that similar discordances occurred among other serotypes between vaccine-serotype carriage prevalence and the post-nadir slope. For example, the carriage prevalence of serotype 6A was 2·1% with a 0·03 post-nadir slope, and serotype 14 had a carriage prevalence of 2·2% with a 0·01 post-nadir slope. We observed a similar discordance in vaccine-serotype carriage prevalence and seroincident events. Serotype 6A showed a nearly two-fold greater prevalence of seroincident events compared with that of serotype 14, despite similar carriage prevalence. This underlines the importance of achieving a better understanding of the serotype-specific mechanism of naturally acquired immunity by cumulative pneumococcal natural exposure.

Recognising that the aggregate 0·35 μg/mL CoP for IPD was derived from pooled infant data limited to high-income (USA) and upper middle-income (South Africa) country settings, regional differences should be considered when evaluating CoPs for carriage and disease,^9^ especially in settings such as Malawi, where vaccine serotypes continue to contribute to pneumococcal carriage and IPD disease burden. Crucially, our data highlight the potential loss of the protective benefit of a 3 + 0 schedule by the second year of life, which, apart from capsule-independent immunity, presents a potential window of vulnerability to carriage and disease. This is particularly relevant given that most sub-Saharan Africa countries implementing PCVs have used primary scheduled with three primary doses without a booster, and they reported similar trends in residual vaccine-serotype carriage.^18^, ^19^ High-income countries implementing PCV without a booster (eg, Australia's previous 3 + 0 schedule with one dose given at ages 2, 4, and 6 months) also reported residual pneumococcal carriage and disease.^32^ Countries implementing a booster dose have observed greater drops in carriage and disease. Additionally, work done in the past few years has underlined the importance of a booster dose in a maintenance schedule (1 + 1), as shown by the UK's recent change to a 1 + 1 schedule^33^ and ongoing evaluations on the African continent and in east Asia.^19^, ^34^

Although this novel population-based analysis was both cost-effective and less complex than a longitudinal cohort study, there were limitations, including the extrapolation of findings to the individual level. First, although we are not aware of a similar population-based study with which we can compare our findings, a similar analysis with similar serological profiles has been done in Thailand.^27^ However, that study was completed before PCV implementation and was limited to children younger than 2 years. Additionally, the interpretation of CoP for pneumococcal carriage remains associated with considerable uncertainty. Further analysis of carriage and serological data from other similar geospatial and temporal settings will strengthen these estimates. Second, the measurement of vaccination status depended predominantly on health passports with little capacity for verification of either recorded or reported vaccination status. However, reported high coverage is concordant to that reported by other studies in Malawi, and any misclassification is thus likely to be small and would not significantly change the findings. Third, carriage CoPs are likely to vary by population depending on several factors, including the force of infection, crowding, and air pollution. We note that the individual IgG measurements were characterised by large variability. This is most likely due to the cross-sectional nature of the data and individual-level differences in IgG driven by demographic, genetic, and environmental variables that were not known to us. Nevertheless, the modelling framework we adopted in this work incorporates this variability in the confidence intervals for model parameters and allowed us to infer population-level average IgG profiles over age.

In conclusion, in the context of a 3 + 0 PCV13 schedule, we report the absence of a sustained vaccine-induced antibody response to multiple vaccine serotypes beyond the first year of life. This might result in a window of vulnerability to both pneumococcal carriage and disease. With evidence of residual vaccine-serotype IPD in settings such as Malawi, including among highly vulnerable neonates,^35^ strategies to counter this residual burden are required to achieve sustained vaccine-induced antibody titres, and thus control. An extended catch-up campaign at the time of PCV introduction can speed up the reduction in vaccine-serotype carriage. Policy decisions should favour including a booster dose later in the first or second year of life, as endorsed by WHO, to achieve sustained vaccine-induced antibody titres. By inducing higher antibody concentrations later in the child's life, the booster dose might lead to longer protection against colonisation and improved indirect protection, which is crucial for successful PCV programmes. However, both the impact and the cost-effectiveness of such strategies need to be evaluated before they are implemented in vaccine programmes.

## Data sharing

Deidentified group data can be available for sharing on application to the corresponding author. This application must include the relevant proposal detailing the intended use of the data and the ethics approval for this proposal, and it requires a signed data sharing agreement. Additional study documents including the study protocol and informed consent form are available on application to the corresponding author on publication of this report.

## Declaration of interests

We declare no competing interests.
