# Supplementary material for: Waning of antibody levels induced by a 13-valent pneumococcal conjugate vaccine, using a 3 + 0 schedule, within the first year of life among children younger than 5 years in Blantyre, Malawi: an observational, population-level, serosurveillance study
Source: Lancet Infect Dis. 2022 Dec;22(12):1737–47. doi: 10.1016/S1473-3099(22)00438-8 (PMC10555849; doi:10.1016/S1473-3099(22)00438-8)
Supplement: Supplementary appendix [file mmc1.pdf]

THE LANCET
Infectious Diseases

Supplementary appendix

This appendix formed part of the original submission and has been peer reviewed. We post it as supplied by the authors.

Supplement to: Swarthout TD, Henrion MYR, Thindwa D, et al. Waning of antibody levels induced by a 13-valent pneumococcal conjugate vaccine, using a 3 + 0 schedule, within the first year of life among children younger than 5 years in Blantyre, Malawi: an observational, population-level, serosurveillance study. *Lancet Infect Dis* 2022; published online Aug 24. https://doi.org/10.1016/S1473-3099(22)00438-8.

Supplementary appendix
Swarthout TD and colleagues

Title: Waning of PCV13 vaccine-induced antibody levels within the first year of life, using a 3+0 schedule: an observational population-level serosurveillance study among children under 5 years old in Blantyre, Malawi.

Table of Contents

Table S1 - Correlates of protection (μg/mL) for invasive pneumococcal disease and carriage ……………………….…………………………. 3
Table S2 - Community carriage serotype distribution among children aged <5 years, PCVPA Study ……………………….……..................... 4
Table S3 - Serotype-specific GMCs calculated using empirical data as shown in manuscript Figure 1, stratified by 3-month age groups …….. 7-8
Table S4 - Assay output summary ……………………………………………………………………………………………………. 9
Table S5 - Trend among paired (primary and secondary) samples, censored for ages <6 months ……………………………………….…... 10
Table S6 - Percent of children attaining CoP thresholds (μg/mL) post vaccination, stratified by age group ……………………….…. 13
Figure S1 - Seroincident events, based on 82 linked pairs …………………………………………………………………................ 11
Figure S2 - Protein-specific antibody profiles, stratified by 3-month age groups ………………………………………………………………… 12
Supplementary methods, linear spline regression model ………………………………………………………………………………… 5-6
References …….. 14

Table S1 - Correlates of protection (µg/mL) for invasive pneumococcal disease and carriage

	IPD		Carriage
	Siber[1] µg/mL	Andrews[2] µg/mL 95%CI	Voysey[3] µg/mL 95%CI
1	0·35	0·78<br>0·47–1·68	0·81<br>0·54–1·22
3	0·35	2·83<br>1·16–∞	
4	0·35	0·35<br>0·20–1·17	1·16<br>0·90–1·49
5	0·35	ND	0·73<br>0·42–1·26
6A	0·35	0·16<br>0·08–1·05 †	
6B	0·35	0·16<br>0·08–2·54	0·50<br>0·40–0·61
7F	0·35	0·87<br>0·40–1·80	1·60<br>1·25–2·05
9V	0·35	0·62<br>0·19–∞	1·31<br>1·08–1·58
14	0·35	0·46<br>0·25–1·12	2·48<br>2·02–3·04
18C	0·35	0·14<br>0·09–0·40	1·32<br>0·95–1·82
19A	0·35	1·00<br>0·60–2·47	
19F	0·35	1·17<br>0·62–4·62	2·54<br>1·83–3·53
23F	0·35	0·20<br>0·08–1·50	0·63<br>0·47–0·83
12F			
33F			

Shaded grey cells represent serotypes that were not evaluated in the respective analyses.

Table S2 - Community carriage serotype distribution among children aged <5 years, PCVPA Study

Serotype	n	Carriage prevalence (%)	Contribution to total VT carriage (%) n=719
VT[§] (n=4 234)			
1	13	0·31	1·81
3	122	2·88	16·97
4	12	0·28	1·67
5	7	0·17	0·97
6A	89	2·10	12·38
6B	27	0·64	3·76
7F	3	0·07	0·42
9V	29	0·68	4·03
14	91	2·15	12·66
18C	16	0·38	2·23
19A	44	1·04	6·12
19F	146	3·45	20·31
23F	120	2·83	16·69
NVT[‡] (n=1 629)			
12F	16	0·98	ND
33F	12	0·73	ND

[§]Source: Pneumococcal carriage surveillance, 2015–2019, latex agglutination (reported in Swarthout TD and colleagues[4]). VTs reported were detected when screening 4 234 nasopharyngeal swab samples for pneumococcal carriage using latex agglutination on cultured samples. [‡]Source: Pneumococcal carriage surveillance, 2015–2017, microarray (reported in Swarthout TD and colleagues[5]). NVTs reported were detected when screening 1 629 nasopharyngeal swab samples for pneumococcal carriage, including by DNA microarray among those culture-confirmed for pneumococcal carriage.

<5 years: includes PCV age-ineligible (pre-vaccine, 4–8 weeks old) and PCV13-vaccinated (3–4 years old). ND=Not done, NVT=non-vaccine serotype, VT= vaccine serotype.

Supplementary methods, linear spline regression models: A flexible modelling framework using linear splines for censored regression models to estimate serological profiles.

Lower limit of detection; censored regression: The assay's lower limit of detection (LLD, 0·15 µg/mL) means that observations below the detection limit (DL) are left-censored. To account for this, we use censored regression. Specifically, we assume a Gaussian linear model for y, the natural logarithm of the measured IgG concentration, with a single explanatory variable, x, age:

$$y = \beta_0 + \beta_1 x + \epsilon = f(x, \boldsymbol{\beta}) + \epsilon \qquad (1)$$

where $\epsilon \sim \mathcal{N}(0, \sigma^2)$.

We do not directly observe y, but only y_M, where values below the detection limit are censored:

$$y_M = \begin{cases} y, & y > \tau \\ \tau, & y \leq \tau \end{cases}$$

where τ is the lower limit of detection ($\tau = 0.15$µg/mL in our data).

Since we assume a Gaussian model, this allows us to write down, for an observed data set $\{x_i, y_{M,i}\}_{i=1}^{n}$, the log-likelihood below[6], thereby making the usual maximum likelihood inference theory available:

$$l(\beta, \sigma^2) = \sum_{y_{M,i} > \tau} \log\left(\phi\left(\left(y_{M,i} - f(x, \boldsymbol{\beta})\right)/\sigma\right)\right) + \sum_{y_{M,i} \leq \tau} \log\left(\Phi(f(x, \boldsymbol{\beta}) - \tau)\right) \qquad (2)$$

Identify and estimate change points; linear regression splines: To address the objectives, we needed to fit a model that allows the linear relationship between age and IgG to change over time. A natural candidate for this is linear splines. Splines fit piece-wise linear regression models, allowing the slope to change at specific values of the predictor variable. These locations, commonly referred to as knots, define intervals over which the relationship between predictor and response is linear.

Mathematically, for a response y and a predictor x, a linear spline with 3 knots at locations $x = k_1, k_2, k_3$ can be written down as:

$$y = \beta_0 + \beta_1 \cdot x + \beta_2 \cdot (x - k_1)_+ + \beta_3 \cdot (x - k_2)_+ + \beta_4 \cdot (x - k_3)_+ + \epsilon \qquad (3)$$

where $(x)_+ = x$ if $x \geq 0$ and 0 otherwise.

This means that the fitted curve has different slopes in different intervals of values of x:

interval	Slope
(-Inf, k_1)	β_1
[k_1,k_2)	$\beta_1 + \beta_2$
[k_2,k_3)	$\beta_1 + \beta_2 + \beta_3$
[k_3,Inf)	$\beta_1 + \beta_2 + \beta_3 + \beta_4$

Statistical inference for number and locations of knots (bootstrap and AIC): Usually splines are used to produce smooth functional fits (typically one would use cubic rather than linear functions) and knot locations are either user-provided or set to regular intervals in the predictor variable.

For the purpose of the pneumococcus serology data however, the number and the locations of the knots, i.e., the change points, are of interest themselves and we would like to do statistical inference on the knot locations. To this end, we treat the knot locations $k_1, k_2, k_3, \ldots$ as model parameters and estimate them using numerical optimisation. We then use the bootstrap to derive confidence intervals for the locations of the knots.

To identify which number of change points best fits the data, we fit models with several numbers of knots, then use Akaike Information Criterion (AIC) to select the optimal number of knots.

Putting it all together: Given the nature of the data (IgG titre with a lower detection limit) and the fact that we consider several plausible biological models for vaccine response profiles (with changing slopes) for which it is important to know when the changes in IgG levels occur, we need our modelling framework to satisfy several requirements:

1. Properly account for data values that are left censored (below the detection limit).
2. Treat the knot locations as parameter values to be estimated during model fitting.
3. Return a fit for the geometric mean IgG concentration as a function of age.
4. Select the optimal number of knots / changepoints.

This means that our model is (1), with $f(x, \boldsymbol{\beta})$ in (1) replaced with $f(x, \boldsymbol{\beta}, \boldsymbol{k})$ using the functional form from (3).

In summary, the key characteristics of our modelling framework are:

1. We fit censored regression models with linear splines for the age predictor variable.
2. Knot locations are treated as model parameters and fitted by numerical minimisation of a least squares function. Since the model is now no longer fitted by maximum likelihood, we use bootstrap techniques to derive confidence intervals for slope parameters (as well as predicted average concentration at any given age) and knot locations. Specifically, the bootstrap percentile method[7] is used for CIs.
3. Before model fitting, the IgG data are log-transformed, so that the fitted arithmetic mean corresponds to the log of the geometric mean on the original data scale (i.e. we can exponentiate the fitted curve to have a fit for the geometric mean).
4. For every serotype, models are fitted with $k = 0,1,2,3$ knots / changepoints and the optimal value for k is selected using AIC.

Our implementation was done in the R environment for statistical programming, making use of packages rms[8] (for the linear spline terms), censReg[11] (for the censored regression likelihood), boot[7,10] (for the bootstrapped CIs) and custom code to estimate model parameters and selecting the number of knots / changepoint.

Conclusion: We present a flexible modelling framework to estimate both the number and location of changepoints in serological profiles. Key benefits of our approach are the principled statistical analysis of serological profile data which are increasingly common and the grouping of profiles, giving insights into the underlying biology.

The modelling code is made available to other researchers on GitHub[11]

Table S3A - Serotype-specific GMCs calculated using empirical data as shown in manuscript Figure 1, stratified by 3-month age groups

**Age band (months)**	n	**Serotype 1**	**Serotype 3**	**Serotype 4**	**Serotype 5**	**Serotype 6A**	**Serotype 6B**	**Serotype 7F**
0–02	12	0·74 (0·30–1·84)	0·41 (0·13–1·24)	0·52 (0·22–1·25)	0·56 (0·30–1·05)	0·31 (0·15–0·66)	0·24 (0·11–0·53)	0·36 (0·15–0·91)
03–05	16	1·27 (0·74–2·16)	0·71 (0·42–1·20)	1·07 (0·55–2·09)	0·93 (0·60–1·45)	0·90 (0·55–1·48)	0·69 (0·36–1·32)	1·49 (0·80–2·76)
06–08	41	0·66 (0·52–0·83)	0·31 (0·25–0·39)	0·47 (0·36–0·62)	0·57 (0·43–0·76)	0·70 (0·50–0·98)	0·70 (0·53–0·92)	0·97 (0·74–1·27)
09–11	49	0·39 (0·32–0·48)	0·28 (0·20–0·41)	0·28 (0·22–0·36)	0·40 (0·33–0·49)	0·58 (0·46–0·73)	0·49 (0·38–0·64)	0·82 (0·65–1·03)
12–14	40	0·24 (0·20–0·28)	0·23 (0·15–0·36)	0·22 (0·17–0·27)	0·44 (0·36–0·54)	0·59 (0·46–0·75)	0·42 (0·34–0·51)	0·60 (0·46–0·78)
15–17	54	0·21 (0·17–0·26)	0·27 (0·18–0·41)	0·15 (0·10–0·22)	0·32 (0·25–0·41)	0·46 (0·36–0·59)	0·37 (0·30–0·45)	0·48 (0·40–0·57)
18–20	43	0·20 (0·15–0·26)	0·20 (0·15–0·27)	0·17 (0·13–0·23)	0·35 (0·28–0·45)	0·51 (0·38–0·68)	0·40 (0·30–0·53)	0·48 (0·35–0·66)
21–23	47	0·19 (0·16–0·24)	0·22 (0·15–0·33)	0·15 (0·10–0·22)	0·38 (0·29–0·50)	0·44 (0·31–0·63)	0·40 (0·29–0·56)	0·40 (0·30–0·52)
24–26	26	0·24 (0·16–0·34)	0·18 (0·09–0·34)	0·11 (0·05–0·23)	0·33 (0·24–0·45)	0·46 (0·29–0·74)	0·40 (0·26–0·62)	0·38 (0·27–0·53)
27–29	18	0·17 (0·09–0·32)	0·16 (0·06–0·38)	0·09 (0·03–0·27)	0·39 (0·26–0·59)	0·77 (0·40–1·49)	0·49 (0·31–0·77)	0·27 (0·18–0·40)
30–32	20	0·23 (0·17–0·32)	0·28 (0·11–0·70)	0·21 (0·14–0·31)	0·48 (0·28–0·81)	0·83 (0·50–1·37)	0·64 (0·41–1·02)	0·33 (0·25–0·43)
33–35	21	0·20 (0·16–0·26)	0·14 (0·06–0·35)	0·22 (0·16–0·30)	0·56 (0·43–0·73)	0·86 (0·58–1·27)	0·61 (0·42–0·90)	0·30 (0·23–0·39)
36–38	20	0·21 (0·16–0·28)	0·16 (0·09–0·30)	0·24 (0·13–0·42)	0·57 (0·39–0·85)	1·16 (0·66–2·02)	0·86 (0·54–1·36)	0·29 (0·24–0·35)
39–41	20	0·16 (0·10–0·25)	0·16 (0·07–0·35)	0·20 (0·12–0·35)	0·61 (0·37–1·00)	1·10 (0·62–1·95)	0·54 (0·34–0·86)	0·30 (0·19–0·48)
42–44	18	0·24 (0·17–0·33)	0·22 (0·12–0·42)	0·29 (0·16–0·52)	0·70 (0·45–1·08)	0·77 (0·43–1·37)	0·52 (0·31–0·88)	0·39 (0·26–0·57)
45–47	21	0·32 (0·19–0·53)	0·33 (0·14–0·76)	0·20 (0·13–0·30)	0·68 (0·47–0·98)	0·97 (0·68–1·37)	0·70 (0·41–1·20)	0·30 (0·23–0·39)
48–50	31	0·32 (0·23–0·44)	0·26 (0·15–0·47)	0·41 (0·24–0·71)	0·86 (0·61–1·20)	1·54 (1·12–2·14)	0·97 (0·68–1·37)	0·31 (0·22–0·43)
51–53	32	0·37 (0·28–0·49)	0·15 (0·07–0·28)	0·35 (0·22–0·53)	0·76 (0·53–1·10)	1·02 (0·73–1·43)	1·04 (0·75–1·43)	0·30 (0·22–0·41)
54–56	18	0·44 (0·29–0·67)	0·35 (0·14–0·87)	0·88 (0·49–1·58)	1·33 (0·90–1·98)	2·10 (1·51–2·92)	1·19 (0·87–1·61)	0·39 (0·26–0·58)
57–59	9	0·24 (0·10–0·58)	0·31 (0·14–0·70)	0·22 (0·10–0·46)	0·70 (0·24–2·04)	2·09 (0·44–9·86)	0·50 (0·27–0·93)	0·35 (0·20–0·61)

Table S3B - Serotype-specific GMCs calculated using empirical data as shown in manuscript Figure 1, stratified by 3-month age group

Age band (months)	Serotype 9V	Serotype 14	Serotype 18C	Serotype 19A	Serotype 19F	Serotype 23F	Serotype 12F	Serotype 33F
0–02	0·41 (0·18–0·95)	1·07 (0·42–2·74)	0·25 (0·11–0·55)	0·63 (0·32–1·23)	0·81 (0·29–2·25)	0·28 (0·10–0·80)	0·05 (0·01–0·37)	0·16 (0·06–0·43)
03–05	0·96 (0·64–1·45)	1·50 (0·78–2·86)	0·78 (0·36–1·70)	1·71 (1·05–2·79)	3·50 (2·26–5·43)	1·09 (0·70–1·70)	0·15 (0·15–0·15)	0·14 (0·09–0·21)
06–08	0·48 (0·36–0·62)	1·78 (1·17–2·71)	0·42 (0·31–0·57)	1·23 (0·93–1·63)	1·37 (1·08–1·73)	0·31 (0·20–0·48)	0·03 (0·00–0·16)	0·07 (0·03–0·15)
09–11	0·34 (0·26–0·45)	1·81 (1·36–2·41)	0·23 (0·17–0·33)	0·71 (0·53–0·96)	0·92 (0·66–1·29)	0·25 (0·19–0·33)	0·15 (0·15–0·15)	0·08 (0·04–0·14)
12–14	0·35 (0·27–0·45)	0·90 (0·64–1·25)	0·18 (0·15–0·23)	0·88 (0·66–1·17)	0·95 (0·69–1·31)	0·21 (0·13–0·34)	0·02 (0·00–0·99)	0·09 (0·05–0·18)
15–17	0·25 (0·18–0·33)	1·08 (0·75–1·55)	0·15 (0·11–0·21)	0·80 (0·54–1·20)	0·81 (0·60–1·09)	0·22 (0·15–0·32)	0·10 (0·06–0·17)	0·07 (0·04–0·13)
18–20	0·28 (0·19–0·40)	0·87 (0·59–1·28)	0·17 (0·11–0·26)	0·94 (0·61–1·44)	0·87 (0·60–1·26)	0·21 (0·12–0·36)	0·03 (0·01–0·13)	0·11 (0·06–0·18)
21–23	0·34 (0·24–0·48)	0·85 (0·60–1·19)	0·14 (0·08–0·23)	0·89 (0·58–1·38)	1·20 (0·82–1·77)	0·20 (0·12–0·35)	0·06 (0·02–0·16)	0·13 (0·08–0·21)
24–26	0·26 (0·16–0·42)	0·64 (0·39–1·03)	0·21 (0·11–0·40)	0·81 (0·48–1·38)	1·03 (0·61–1·74)	0·33 (0·17–0·62)	0·04 (0·00–0·59)	0·13 (0·08–0·21)
27–29	0·18 (0·09–0·37)	0·68 (0·35–1·34)	0·21 (0·10–0·44)	1·15 (0·65–2·03)	1·08 (0·59–1·96)	0·11 (0·03–0·38)	0·15 (0·15–0·15)	0·13 (0·05–0·31)
30–32	0·32 (0·21–0·49)	1·88 (1·06–3·31)	0·19 (0·12–0·29)	1·25 (0·66–2·39)	1·52 (0·77–2·99)	0·23 (0·16–0·33)	0·03 (0·00–0·37)	0·13 (0·06–0·30)
33–35	0·44 (0·32–0·60)	1·21 (0·64–2·31)	0·21 (0·13–0·33)	2·83 (1·55–5·16)	1·76 (1·06–2·93)	0·25 (0·12–0·52)	0·06 (0·01–0·23)	0·13 (0·07–0·24)
36–38	0·53 (0·27–1·04)	0·81 (0·39–1·71)	0·19 (0·09–0·43)	2·11 (1·37–3·25)	2·12 (1·18–3·81)	0·23 (0·12–0·42)	0·14 (0·10–0·18)	0·11 (0·04–0·35)
39–41	0·32 (0·20–0·52)	1·25 (0·69–2·28)	0·25 (0·12–0·50)	3·04 (1·36–6·77)	2·18 (1·21–3·92)	0·21 (0·10–0·47)	0·05 (0·01–0·30)	0·18 (0·10–0·30)
42–44	0·44 (0·28–0·69)	1·08 (0·64–1·83)	0·26 (0·17–0·40)	1·49 (0·89–2·47)	1·65 (0·97–2·79)	0·41 (0·25–0·65)	0·09 (0·04–0·20)	0·16 (0·09–0·30)
45–47	0·48 (0·35–0·65)	1·30 (0·77–2·18)	0·18 (0·12–0·27)	2·19 (1·36–3·51)	1·41 (0·90–2·21)	0·28 (0·13–0·59)	0·01 (0·00–0·97)	0·14 (0·08–0·25)
48–50	0·81 (0·59–1·12)	1·12 (0·72–1·75)	0·41 (0·24–0·68)	2·44 (1·61–3·71)	2·45 (1·72–3·49)	0·49 (0·31–0·78)	0·04 (0·00–0·26)	0·19 (0·13–0·27)
51–53	0·58 (0·41–0·81)	1·36 (0·89–2·07)	0·48 (0·30–0·76)	2·47 (1·72–3·55)	2·21 (1·61–3·05)	0·44 (0·28–0·67)	0·06 (0·02–0·23)	0·28 (0·17–0·47)
54–56	0·94 (0·65–1·37)	2·01 (0·98–4·10)	0·61 (0·36–1·03)	4·97 (3·08–8·02)	3·52 (2·24–5·52)	0·46 (0·31–0·68)	0·24 (0·11–0·54)	0·33 (0·21–0·50)
57–59	0·66 (0·24–1·79)	0·94 (0·59–1·51)	0·28 (0·10–0·74)	2·84 (1·15–6·99)	3·57 (0·94,13·65)	0·28 (0·07–1·10)	0·32 (0·10–1·02)	0·58 (0·25–1·37)

Table S4 - Assay output summary for analysis of primary samples.

Serotype	< LLD[¶] n (%)	NR[§] N (%)	QNS[ǂ] n (%)	In analysis of primary samples n (%)		~~In analysis of secondary samples, n pairs~~	Maximum titre (median) µg/mL
1	132 (20·7)	22 (3·5)	1 (0·2)	615 (96·4)	~~534~~	~~72~~	6·63 (0·25)
3	202 (31·7)	36 (5·6)	3 (0·5)	599 (93·9)	~~519~~	~~70~~	8·04 (0·24)
4	217 (34·0)	0 (0·0)	2 (0·3)	636 (99·7)	~~554~~	~~76~~	9·64 (0·24)
5	52 (8·2)	3 (0·5)	1 (0·2)	634 (99·4)	~~553~~	~~74~~	6·72 (0·47)
6A	43 (6·7)	0 (0·0)	1 (0·2)	637 (99·8)	~~555~~	~~76~~	24·55 (0·66)
6B	62 (9·7)	3 (0·5)	1 (0·2)	634 (99·4)	~~552~~	~~76~~	22·66 (0·50)
7F	55 (8·6)	0 (0·0)	3 (0·5)	635 (99·5)	~~553~~	~~75~~	8·66 (0·43)
9V	112 (17·6)	9 (1·4)	2 (0·3)	627 (98·3)	~~546~~	~~72~~	15·49 (0·40)
14	30 (4·7)	2 (0·31)	1 (0·2)	635 (99·5)	~~554~~	~~75~~	63·49 (1·13)
18C	219 (34·3)	11 (1·7)	2 (0·3)	625 (98·0)	~~546~~	~~71~~	11·92 (0·24)
19A	19 (3·0)	0 (0·0)	1 (0·2)	637 (99·8)	~~555~~	~~76~~	71·31 (1·19)
19F	9 (1·4)	4 (0·6)	2 (0·3)	632 (99·1)	~~552~~	~~74~~	69·28 (1·29)
23F	204 (32·0)	7 (1·10)	3 (0·5)	628 (98·4)	~~547~~	~~73~~	12·90 (0·27)
12F	467 (73·2)	108 (16·9)	2 (0·3)	528 (82·8)	~~455~~	~~64~~	3·37 (LLD)
33F	348 (54·6)	7 (1·10)	1 (0·2)	630 (98·7)	~~549~~	~~75~~	11·02 (LLD)
PsaA	0	0	1	637 (98·7)	~~555~~	-	13846·96
NanA	0	0	1	637 (98·7)	~~554~~	-	8842·46
Ply	0	1	1	636 (98·7)	~~555~~	-	3873·81

[¶]<LLD (less than the lower limit of detection): refers to a value below the assay's lower limit of detection. Samples <LLD do have detectable IgG, but the ELISA assay for IgG quantification cannot accurately determine the concentration value. The LLD was <0·150 µg/mL. [§]NR (no result): ELISA sample results that were non-parallel were then retested. If the retest was also non-parallel, then a result of NR (no result) was recorded. [ǂ]QNS (quantity not sufficient): There was inadequate volume of sample to measure all targeted antibodies. [ǀ]In analyses of primary samples=[638-(NR+QNS)].

Table S5 - Trend among paired (primary and secondary) samples, censored for ages >6 months

**IgG trend**	**1**<br>**% (n)**	**3**<br>**% (n)**	**4**<br>**% (n)**	**5**<br>**% (n)**	**6A**<br>**% (n)**	**6B**<br>**% (n)**	**7F**<br>**% (n)**	**9V**<br>**% (n)**	**14**<br>**% (n)**	**18C**<br>**% (n)**	**19A**<br>**% (n)**	**19F**<br>**% (n)**	**23F**<br>**% (n)**	**12F**<br>**% (n)**	**33F**<br>**% (n)**
ncrease ≥4-fold[§]	4·1 (3)	8·5 (6)	5·2 (4)	4·0 (3)	2·6 (2)	5·2 (2)	1·3 (1)	5·5 (4)	6·6 (5)	4·2 (3)	10·5 (8)	4·0 (3)	8·2 (6)	0·0 (0)	2·6 (2)
Increase ≥2-fold[¶]	12·5 (9)	17·1 (12)	15·7 (12)	10·8 (8)	15·7 (12)	10·5 (8)	4· 0(3)	15·2 (11)	10·6 (8)	11·2 (8)	18·4 (14)	13·5 (10)	16·4 (12)	3·1 (2)	9·3 (7)
Increase <2-fold	6·9 (5)	18·5 (13)	11·8 (9)	37·8 (28)	22·3 (17)	21·0(16)	16·0 (12)	15·2 (11)	9·3 (7)	9·8 (7)	27·6 (21)	25·6 (19)	10·9 (8)	1·5 (1)	9·3 (7)
Any increase, %	19·4	35·6	27·5	48·6	38·0	31·5	20·0	30·4	19·9	21·0	46·0	39·1	27·3	4·6	18·6
No change	0·0 (0)	0·0 (0)	0·0 (0)	0·0 (0)	0·0 (0)	0·0 (0)	0·0 (0)	0·0 (0)	1·3 (1)	1·4 (1)	0·0 (0)	0·0 (0)	0·0 (0)	0·0 (0)	0·0 (0)
LLD at V1 & V2	12·5 (9)	17·1 (12)	25·0 (19)	5·4 (4)	5·2 (4)	5·2 (4)	4·0 (3)	16·6 (12)	1·3 (1)	23·9 (17)	1·3 (1)	0·0 (0)	31·5 (23)	84·3 (54)	48·0 (36)
Decrease	68·0 (49)	47·1 (33)	47·3 (36)	45·9 (34)	56·5 (43)	63·1 (48)	76·0 (57)	52·7 (38)	77·3 (58)	53·5 (38)	52·6 (40)	60·8 (45)	41·1 (30)	10·9 (7)	33·3 (25)
Total pairs in analysis, N[¥]	72	70	76	74	76	76	75	72	75	71	76	74	73	64	75

[¶]Increase ≥2-fold includes all samples with increase ≥4-fold; [§]Increase ≥4-fold may include all or a subset of those with increase ≥2-fold.
[¥]Though 82 pairs were collected and sent for laboratory analysis, some samples had results that excluded them from this analysis (incl., inadequate volume, no IgG detected).
LLD = lower limit of detection of assay (0·15μg/mL). VT=vaccine serotype, NVT=non-vaccine serotype, V1=visit 1 including primary sample collection, V2=visit 2 including secondary sample collection, n=number of paired samples meeting the defined IgG trend

Table S5 reports the trend in paired samples, showing either a decrease, an increase or no change in IgG titre when comparing primary and secondary samples. We hypothesise that an increase in IgG titre is evidence of a novel pneumococcal acquisition event after the primary sample and before the secondary sample, resulting in an increase in serotype-specific IgG. An alternative explanation for an increase in IgG is a novel acquisition event occurring before the primary sample but with an increased IgG (relative to the primary sample) still detected at secondary sample. A decrease in IgG titre is evidence of no novel pneumococcal acquisition event after the primary sample. Samples with LLD at both visits had detectable IgG too low to quantify.

Method of calculation

Step 1: Calculation of the number of samples meeting the specified criteria (eg, increase ≥ 4-fold) per serotype: On an individual level, divide the serotype-specific lgG titre of the secondary sample by the serotype-specific lgG titre of the primary sample. Then, count the number of individuals meeting the specified criteria (eg, quotient ≥ 4).

Step 2: Calculation of the percentage of samples meeting the specified criteria (eg, increase ≥ 4-fold) per serotype: Divide the number of samples meeting the specified criteria per serotype by the total number of paired samples available for the serotype. Then, multiply the quotient by 100%.
Procedure explained above (Step1 and Step 2) was repeated for all serotypes and specified criteria.

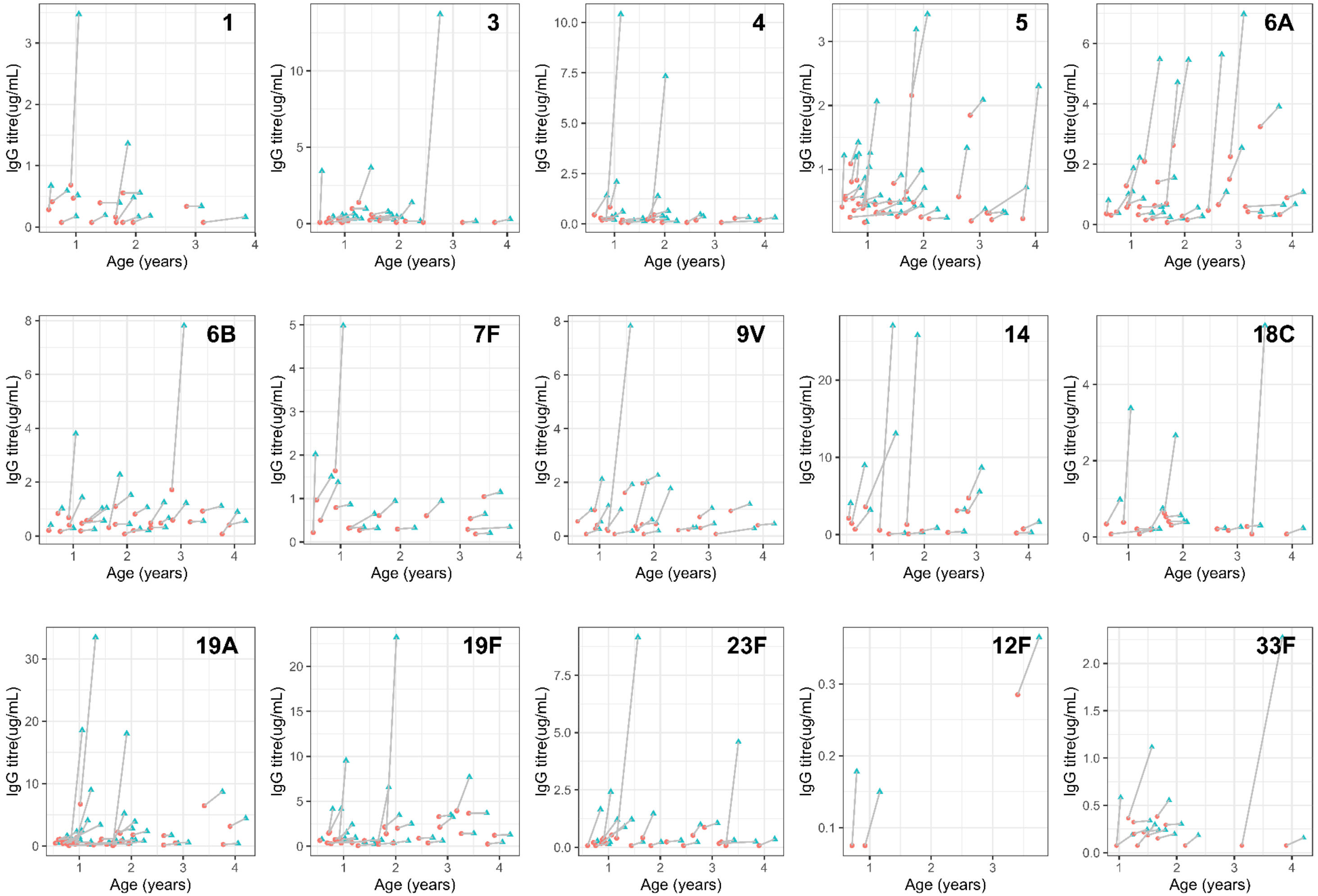

Figure S1 - Seroincident events, based on 82 linked pairs (limited to samples with increase in IgG titres)

Figure S1 reports the seroincident events based on 82 linked pairs with the respective secondary serum samples collected approximately 3 months after the primary samples. Data reported are censored to include only children at least 6 months of age and limited to pairs with any increase in IgG titres (positive slope). Red dot=primary sample. Blue triangle=secondary sample.

2A. PsaA

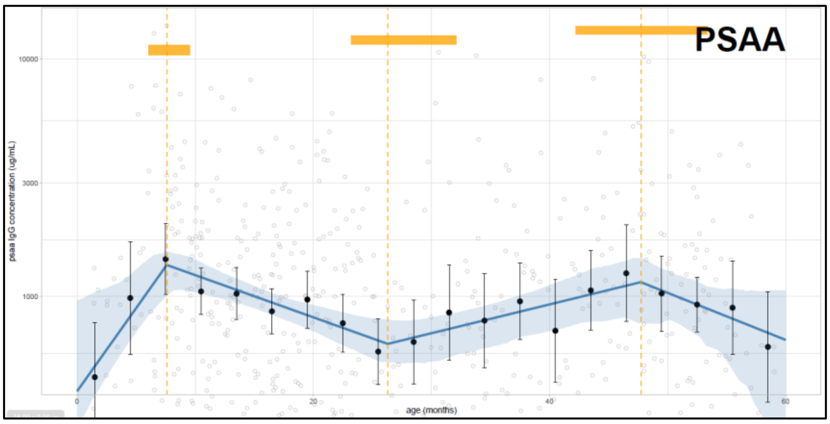

2B. NanA

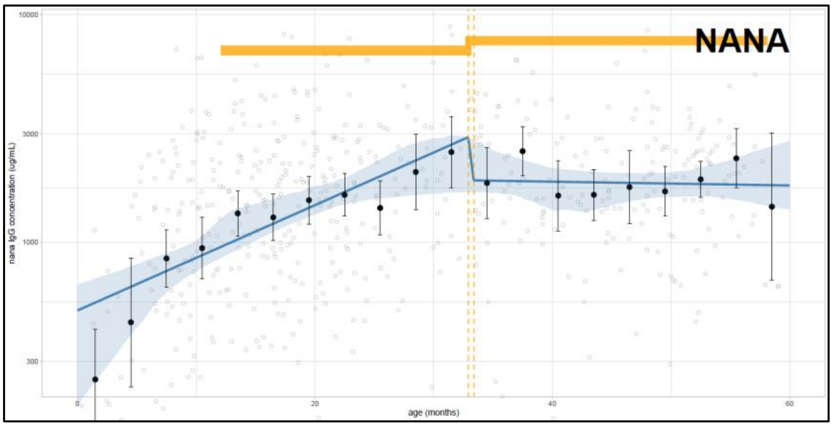

2C. Ply

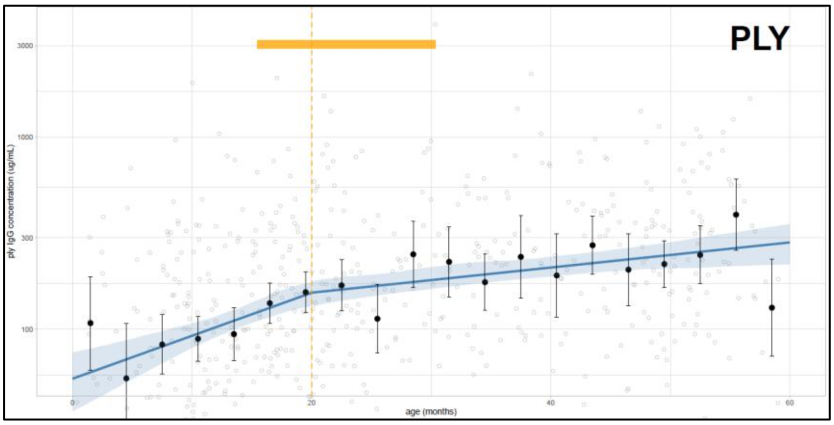

Figure S2 - Protein-specific antibody profiles, stratified by 3-month age groups. Grey dots indicate empirical IgG titre datapoints for each sample. Black dots (with 95% confidence intervals [CIs] are the geometric mean concentrations (GMCs) for all datapoints within 3-month age groups. The blue line is the spline model fit and the blue shaded area is the 95% CI for the model fit. Vertical dashed lines indicate changepoints. Orange bars at the top of each image are the 95% CI for the age at each changepoint. PsaA=Pneumococcal surface adhesin A. NanA=Neuraminidase A. Ply=pneumolysin.

Table S6. Percent of children attaining CoP thresholds (µg/mL) post vaccination, stratified by age group

Age	Serotype 1 (n= 534)			Serotype 3 (n=519)			Serotype 4 (n=554)			Serotype 5 (n=553)		
	≥ 0·35[¥]	≥ CoPd[¶]	≥ CoPc[§]	≥ 0.35[¥]	≥ CoPd[¶]	≥ CoPc[§]	≥ 0·35[¥]	≥ CoPd[¶]	≥ CoPc[§]	≥ 0·35[¥]	≥ CoPd[¶]	≥ CoPc[§]
**<3m**	58·3	50·0	50·0	41·6	16·6	-	58·3	-	25·0	58·3	-	41·6
**3-5m**	87·5	81·3	81·3	73·3	6·6	-	75·0	-	56·2	87·5	-	56·2
**6-11 m**	67·4	28·1	25·8	40·0	1·1	-	51·6	-	12·0	61·1	-	31·1
**1y**	20·3	3·4	2·8	36·9	3·4	-	23·6	-	2·1	49·7	-	23·2
**2y**	26·5	4·8	4·8	36·2	7·5	-	25·0	-	2·3	58·3	-	30·9
**3y**	22·7	6·7	6·7	33·8	2·8	-	36·0	-	10·6	69·7	-	40·7
**4y**	48·8	13·4	13·4	35·0	3·7	-	56·6	-	25·5	81·1	-	55·5
**Total**	36·7	13·1	12·6	37·7	4·1	-	37·7	-	10·8	62·0	-	34·7

Age	Serotype 6A (n=555)			Serotype 6B (n=552)			Serotype 7F (n=553)			Serotype 9V (n=546)		
	≥ 0·35[¥]	≥ CoPd[¶]	≥ CoPc[§]	≥ 0·35[¥]	≥ CoPd[¶]	≥ CoPc[§]	≥ 0·35[¥]	≥ CoPd[¶]	≥ CoPc[§]	≥ 0·35[¥]	≥ CoPd[¶]	≥ CoPc[§]
**<3m**	41·6	66·6	-	25·0	66·6	25·0	41·6	33·3	16·6	45·4	36.3	18.1
**3-5m**	87·5	93·7	-	68·7	87·5	43·7	81·2	68·7	56·2	93·7	75.0	43.7
**6-11 m**	73·6	92·3	-	73·6	92·3	60·4	85·7	50·5	25·2	56·6	25.5	10.0
**1y**	59·6	90·8	-	54·6	86·8	35·5	62·1	21·0	10·8	43·6	22.6	8.2
**2y**	73·8	95·2	-	69·0	96·4	44·0	46·4	8·3	3·5	46·3	19.5	6.1
**3y**	80·2	90·7	-	72·3	86·8	56·5	36·8	10·5	2·6	55·2	32.8	15.7
**4y**	92·2	100	-	85·6	97·7	75·5	46·0	13·4	4·4	78·8	60.0	24.4
**Total**	72·6	92·7	-	67·2	90·5	50·3	57·6	22·9	11·3	55·1	32.0	13.1

Age	Serotype 14 (n= 554)			Serotype 18C (n= 546)			Serotype 19A (n=555)			Serotype 19F (n=552)		
**<3m**	75·0	66·6	33·3	25·0	75·0	16·6	66·6	66·6	-	83·3	25.0	25.0
**3-5m**	87·5	81·2	43·7	68·7	81·2	50·0	100·0	100	-	100	93.	68.7
**6-11 m**	86·8	82·4	43·9	49·4	71·9	8·9	80·2	8·9	-	92·1	39.3	13.4
**1y**	79·0	73·1	17·2	20·2	52·4	3·2	73·6	8·4	-	77·9	36.0	18.8
**2y**	77·3	66·6	25·0	26·5	65·0	8·4	82·1	82·1	-	86·7	55.4	26.5
**3y**	82·6	73·3	26·6	32·8	63·0	5·4	89·4	89·4	-	92·1	71.0	36.8
**4y**	87·7	81·1	25·5	57·7	78·8	16·6	96·6	96·6	-	98·8	78.8	45.5
**Total**	82·1	75·0	26·5	35·3	64·6	9·1	82·5	82·5	-	87·6	52.7	27.5

Age	Serotype 23F (n=554)			Serotype 12F (n=455)			Serotype 33F (n=455)		
**<3m**	50.0	66.6	41.6	8·3	-	-	8·3	-	-
**3-5m**	87.5	100.0	68.7	0·0	-	-	0·0	-	-
**6-11 m**	35.9	60.6	21.3	1·1	-	-	1·1	-	-
**1y**	36.2	48.6	21.6	1·1	-	-	1·1	-	-
**2y**	37.3	55.4	22.8	5·9	-	-	5·9	-	-
**3y**	40.5	58.1	27.0	1·8	-	-	1·8	-	-
**4y**	60.2	73.8	36.3	19·2	-	-	19·2	-	-
**Total**	42.6	58.8	26.6	4·1	-	-	4·1	-	-

[¥]Aggregate CoP for IPD (Siber and colleagues)[7], [¶]Putative CoP for disease (Andrews and colleagues)[8]: serotype 1=0·78, 3=2·83, 4=0·35, 5=undefined, 6A=0·16, 6B=0·16, 7F=0·87, 9V=0·62, 14=0·46, 18C=0·14, 19A=1·00, 19F=1·17, 23F=0·20; [§]Putative correlate of protection for carriage (Voysey and colleagues)[11]: serotype 1=0·81, 3=undefined, 4=1·16, 5=0·73; 6A=undefined, 6B=0·50, 7F=1·60, 9V=1·31, 14=2·48, 18C=1·32, 19A=undefined, 19F=2·54, 23F=0·63. Dash (-) in cell indicates that the CoP is undefined. m=month, y=year

References

1. Siber GR, Chang I, Baker S, et al. Estimating the protective concentration of anti-pneumococcal capsular polysaccharide antibodies. Vaccine 2007; 25(19): 3816-26.
2. Andrews NJ, Waight PA, Burbidge P, et al. Serotype-specific effectiveness and correlates of protection for the 13-valent pneumococcal conjugate vaccine: a postlicensure indirect cohort study. *Lancet Infect Dis* 2014; 14(9): 839-46.
3. Voysey M, Fanshawe TR, Kelly DF, et al. Serotype-Specific Correlates of Protection for Pneumococcal Carriage: An Analysis of Immunity in 19 Countries. *Clin Infect Dis* 2018; 66(6): 913-20.
4. Swarthout TD, Fronterre C, Lourenco J, et al. High residual carriage of vaccine-serotype Streptococcus pneumoniae after introduction of pneumococcal conjugate vaccine in Malawi. *Nat Commun* 2020; **11**(1): 2222
5. Swarthout TD, Gori A, Bar-Zeev N, et al. Evaluation of pneumococcal serotyping in nasopharyngeal carriage isolates by latex agglutination, whole genome sequencing (PneumoCaT) and DNA microarray in a high pneumococcal carriage prevalence population in Malawi. *J Clin Microbiol* (2020); 02103-20. https://doi.org/10.1128/JCM.02103-20
6. Kleiber C. and Zeileis A (2008) *Applied Econometrics with R*, Springer, New York, ISBN 978-0-387-77316-2
7. Davison, AC and Hinkley, DV (1997), *Bootstrap Methods and Their Applications*, Cambridge University Press, Cambridge, ISBN 0-521-57391-2
8. Harrell, FE Jr (2021) *rms: Regression Modeling Strategies*, R package version 6.2-0, https://CRAN.R-project.org/package=rms
9. Henningsen A (2020) *censReg: Censored Regression (Tobit) Models*, R package version 0.5-32, https://CRAN.R-project.org/package=censReg
10. Canty A and Ripley B (2021) *boot: Bootstrap R (S-Plus) Functions*, R package version 1.3-28, https://CRAN.R-project.org/package=boot
11. Henrion, MYR (2022) https://github.com/gitMarcH/PCVPA_IgGCensoredRegressionSplineModelling
